# Diet and Physical Activity as Determinants of Continuously Measured Glucose Levels in Persons at High Risk of Type 2 Diabetes

**DOI:** 10.3390/nu14020366

**Published:** 2022-01-15

**Authors:** Su Hyun Park, Jiali Yao, Xin Hui Chua, Suresh Rama Chandran, Daphne S. L. Gardner, Chin Meng Khoo, Falk Müller-Riemenschneider, Clare Whitton, Rob M. van Dam

**Affiliations:** 1 Saw Swee Hock School of Public Health, National University of Singapore and National University Health System, Singapore 117549, Singapore; suhyun.park@nus.edu.sg (S.H.P.); ephyaoj@nus.edu.sg (J.Y.); xinhui.chua@nus.edu.sg (X.H.C.); falk.m-r@nus.edu.sg (F.M.-R.); 2 Department of Endocrinology, Singapore General Hospital, Singapore 169608, Singapore; suresh.rama.chandran@singhealth.com.sg (S.R.C.); daphne.gardner@singhealth.com.sg (D.S.L.G.); 3 Department of Medicine, Yong Loo Lin School of Medicine, National University of Singapore, 14 Medical Drive, Singapore 117599, Singapore; mdckcm@nus.edu.sg; 4 Department of Medicine, National University Health System, Singapore 119228, Singapore; 5 Digital Health Center, Berlin Institute of Health, Charite University Medical Center, 10178 Berlin, Germany; 6 School of Public Health, Curtin University, Perth 6102, Australia; 7 Department of Exercise and Nutrition Sciences, Milken Institute School of Public Health, The George Washington University, Washington, DC 20052, USA

**Keywords:** glucose variability, dietary intake, accelerometry, glucose monitor, mobile phone, physical activity, sedentary behavior

## Abstract

We examined how dietary and physical activity behaviors influence fluctuations in blood glucose levels over a seven-day period in people at high risk for diabetes. Twenty-eight participants underwent a mixed meal tolerance test to assess glucose homeostasis at baseline. Subsequently, they wore an accelerometer to assess movement behaviors, recorded their dietary intakes through a mobile phone application, and wore a flash glucose monitoring device that measured glucose levels every 15 min for seven days. Generalized estimating equation models were used to assess the associations of metabolic and lifestyle risk factors with glycemic variability. Higher BMI, amount of body fat, and selected markers of hyperglycemia and insulin resistance from the meal tolerance test were associated with higher mean glucose levels during the seven days. Moderate- to vigorous-intensity physical activity and polyunsaturated fat intake were independently associated with less variation in glucose levels (CV%). Higher protein and polyunsaturated fatty acid intakes were associated with more time-in-range. In contrast, higher carbohydrate intake was associated with less time-in-range. Our findings suggest that dietary composition (a higher intake of polyunsaturated fat and protein and lower intake of carbohydrates) and moderate-to-vigorous physical activity may reduce fluctuations in glucose levels in persons at high risk of diabetes.

## 1. Introduction

The prevalence of type 2 diabetes mellitus (T2DM) has increased rapidly in many countries, imposing a large health burden on individuals and societies with high medical costs and productivity loss [1]. In 2019, an estimated 463 million adults (9.3%) were living with diabetes worldwide, and this number is projected to increase to 578 million (10.2%) by 2030 [2]. Variation in blood glucose throughout the day provides valuable information not fully captured by HbA1c [3,4]. Given that large fluctuations in blood glucose concentrations may be detrimental to the risk of T2DM and cardiovascular diseases [5,6,7,8], understanding the determinants of these fluctuations may inform preventive interventions.

Over the past decade, the use of continuous glucose monitoring (CGM) devices has enabled a more detailed assessment of the glycemic variation by measuring the duration, frequency, and magnitude of glucose fluctuations, including hypo- and hyperglycemic episodes [9]. Plasma glucose concentrations may fluctuate substantially depending on physical activity [10,11], sleep [12], sedentary behaviors [13,14], and dietary factors [15] in persons with diabetes. For example, a systematic review of 10 randomized controlled trials in persons with T2DM suggested that physical activity reduces glucose variability [10]. In a recent experimental study in persons with T2DM, more sedentary time was significantly associated with less time in the target glucose range, whereas the opposite effect was found for breaks in sedentary time [13]. However, few studies have examined the lifestyle determinants of fluctuations in blood glucose concentrations in persons without diabetes. One trial demonstrated that interrupting sitting reduced postprandial glucose levels after the first and second meal in healthy adolescents [16]. In another trial, a diet with low glycemic index reduced 24 h glucose levels in 10 healthy young individuals [17]. These studies did not simultaneously consider different lifestyle behaviors, which is required to better understand their relative importance for variation in glucose levels.

A traditional epidemiological approach in research on lifestyle and health is to assess usual behaviors during a previous period using questionnaires. However, this approach is prone to recall bias and does not capture the variation in real-life contexts [18]. Accelerometers provide an opportunity for continuous monitoring of movement behaviors, including physical activity, sedentary behavior, and sleep. Furthermore, recording dietary intakes using mobile phone applications has the advantage that it does not rely on participant recall, as exposures are recorded at or near to the time of occurrence [19,20]. Assessment of diet and activity behaviors in real-time thus seems an appropriate approach to understanding plasma glucose fluctuations throughout the day. 

We evaluated how dietary intakes and movement behaviors (i.e., physical activity, sedentary time, sleep) influence glycemic variability in persons at high risk of type 2 diabetes. We measured dietary intake in real-time using mobile phones, movement behaviors with an accelerometer, metabolic measures using a mixed meal tolerance test, and glycemic variability using a glucose monitoring device.

## 2. Materials and Methods

### 2.1. Study Participants

Participants aged 21 to 60 years were recruited from the Singapore Multi-Ethnic Cohort (MEC), a population-based cohort of Singapore citizens and permanent residents aged 21 years and older [21]. Participants were contacted by telephone by a trained research assistant who explained the study. Interested participants underwent a telephone screening and made an appointment for the first study visit. The inclusion criteria included having HbA1c values of 6.0–6.4%, a history of diabetes in first-degree family members (any type), or a body mass index (BMI) of 25 to 30 kg/m^2^. Exclusion criteria were having known sensitivity to medical-grade adhesives, allergies to ingredients of the study meal, pregnancy or lactation, long-term aspirin use, and self-reported medical conditions (bleeding disorders, diabetes mellitus of any type, hypertension, thyroid disease, myocardial infarction, cancer, psychosocial impairment). All participants provided informed consent before participating in the study. 

### 2.2. Study Design

**Figure 1:**
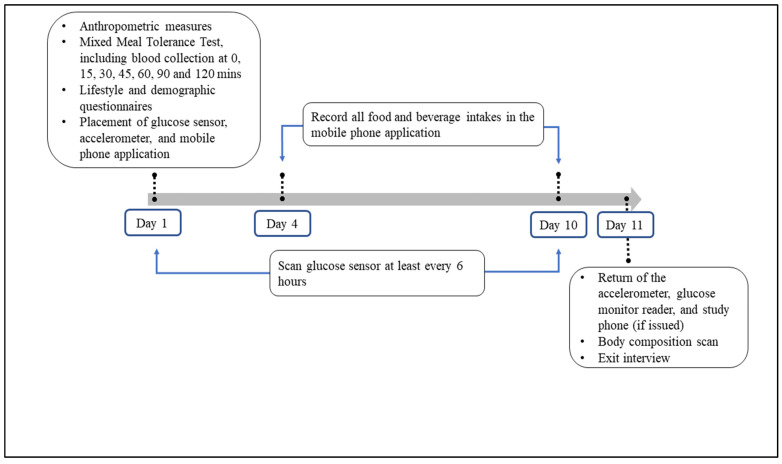
Study schedule.

Figure 1 outlines the study schedule. This observational study consisted of a baseline visit (day 1) and follow-up visit (day 11 or later) at the National University Hospital Singapore and a free-living study period in between. The baseline visit involved a mixed meal tolerance test, anthropometric measurements, demographic and lifestyle questionnaires, and the application of a glucose monitoring sensor (FreeStyle Libre^TM^, Abbott Diabetes Care, Witney, UK) and an accelerometer (Actigraph − Model wGT3x + BT). The glucose monitoring sensor and accelerometer were used to measure objective glucose and movement during the 10-day study period. Participants also received a smartphone application (Jana Care Inc., Boston, MA, USA) to record their food and beverage consumption during days 4 to 10 of the study. During the follow-up visit, body composition was measured, study devices were returned, and an exit interview was conducted. This study was approved by the National Healthcare Group Domain Specific Review Board (NHG DSRB, Ref: 2018/01220), Singapore.

#### 2.2.1. Baseline Assessments 

Participants were instructed to refrain from strenuous exercise for 48 h and consuming alcohol for 24 h, and to fast overnight for 10 to 12 h before the baseline visit. They were also asked to refrain from taking Vitamin C, multivitamins, and aspirin during the 11-day study period. Weight and height were measured using a stadiometer (Seca 284 Digital Measuring Station, Hamburg, Germany), in light clothing without footwear. Blood pressure was measured (Omron HEM-7121, Japan) after resting for five minutes. A standard mixed meal tolerance test (MMTT) [22] was performed after taking the baseline blood sample. The standard MMTT consisted of fried rice which consisted of cooked long grain rice with eggs (Egg Rice, Tesco brand, NTUC Fair Price Co-operative Ltd., Singapore), with each serving containing 50 g carbohydrates, 6.1 g fat, 6.8 g protein, and a total weight of 169.5 g (1.73 kcal/g). 

After participants finished the meal, blood samples were collected at 15, 30, 45, 60, 90, and 120 min for blood glucose and insulin measurements. Insulin sensitivity was assessed via the Matsuda index using the fasting and mean glucose and insulin measurements at 30, 60, 90, and 120 min after the meal (Matsuda index = 10,000/(*G*_fasting_ × *I*_fasting_ × *G*_mean_ × *I*_mean_)^1/2^ [23]. HOMA2-IR (insulin resistance) was calculated using fasting glucose and insulin [24]. The incremental Areas Under the Curve (iAUC) for glucose and insulin responses were calculated using the trapezoidal rule [25]. The early-phase insulin response was assessed by the insulinogenic index as the ratio of the insulin level at 30 min minus fasting insulin to the difference of glucose levels at the same time-points [26]. The oral disposition index (DI), a measure of beta-cell function relative to insulin sensitivity, was calculated as a product of the Matsuda index and insulin secretion index [27]. The insulin secretion index was calculated using the ratio of total AUC insulin (mU/L·min) to total AUC glucose (mmol/L·min) [26]. Questionnaires were administered to collect demographic characteristics.

#### 2.2.2. Follow-Up Assessments

Participants attended the follow-up visit 11 to 20 (median 11) days after the baseline visit. Participant body fat percentage was measured using air displacement plethysmography (Bod Pod, COSMED USA, Inc., Concord, CA, USA), and body fat mass (kg) and fat-free mass (kg) were calculated. 

### 2.3. Monitoring of Lifestyle and Glucose Levels during the Free-Living Period

#### 2.3.1. Glucose Monitoring 

A flash glucose monitoring sensor (FreeStyle Libre^TM^, Abbott Diabetes Care, Witney, UK), the personal version, was applied to the mid-upper part of participants’ upper (non-dominant) arm during the baseline visit. The sensor was part of a flash glucose monitoring system and measured time-stamped glucose levels every 15 min. The sensor was activated with the paired portable reader device after the first hour of wear, and participants were asked to wear the sensor continuously until the follow-up visit. Participants were instructed to scan the sensor with the reader device every 6 h during waking hours to transfer the data for future download as the sensor can store data for up to 8 h. The blood glucose reading on the reader screen was masked to prevent participants from reading glucose values and modifying their usual behavior. Four participants experienced sensor dislodgement and had another sensor fitted and repeated the monitoring period. The refitting for sensor dislodgement was not needed if five days of complete data were obtained from the sensor. Because data from this monitoring system is most accurate after the first 24 h of wear [28] and the sensor was fitted at around noon of day 1, we decided to take a cautionary approach and exclude the first 48 h of wear time. For ease of interpretation, we used whole days of data, beginning with day 4. Days with at least 16 h of measurement were included in analyses, and the number of valid days (≥16 h) ranged from 3 to 7 days (mean 6.9, SD 1.3). We calculated the following metrics as the study outcome variables: mean glucose, coefficient of variation (CV, %) of the daily glucose values (mmol/L), and average daily percentages of time with glucose values above range (>7.8 mmol/L), below range (<3.0 mmol/L), and in range (≥3.0 to ≤7.8 mmol/L) [29]. The selected indices were suggested to be core CGM metrics in clinical practice, according to an international consensus by the Advanced Technologies and Treatments for Diabetes Congress in 2019 [30].

#### 2.3.2. Assessment of Dietary Intake

A mobile phone application (‘the app’) was developed for this study (Jana Care Inc., Boston, MA, USA), adapted from a coaching app used in another study. Prior to that study, the food logging element underwent usability testing as part of that study, as part of several rounds of qualitative evaluations of the acceptability of the app among end-users. The app included a food diary logging system. The food diary involved selecting a meal type (i.e., breakfast, lunch, dinner, or snack), followed by a free text search to find a matching food or beverage. Once a matching item was chosen, participants selected the corresponding portion size. A database of foods commonly eaten in Singapore was incorporated into the app [31], along with an extensive US food database [32]. Participants were also able to re-select foods and beverages that they had already reported, which were displayed under the search bar, without conducting a new search. A handout including images of various cups, plates, and quantity conversions was provided to participants to facilitate portion size estimation. All participants were required to install the app onto their mobile phones. All participants were issued a study phone (Samsung Galaxy S7, Android OS 7.0) as their phones were not compatible. After a training session with the interviewer, participants were required to log their food and beverage intake from day four until the follow-up study visit. Participants received an SMS on day 3 of the study reminding them to commence logging the following day. Reported consumption data were downloaded from the app and merged with nutrient profiles for each consumed food or beverage to calculate energy and nutrient intakes. Participant days with implausible energy intakes (<500 kcal or >5000 kcal) were excluded from the analyses. We calculated intakes of total carbohydrate, protein, saturated fat, monounsaturated fatty acids (MUFA), polyunsaturated fatty acids (PUFA), and fiber. The available carbohydrate was highly correlated with the total carbohydrate (Pearson correlation coefficient: 0.989), therefore it was not investigated separately. Macronutrients were expressed as a percentage of total energy intake, and fiber intake was expressed as grams per 1000 kcal.

#### 2.3.3. Accelerometry Assessment

During the baseline visit, participants were fitted with an accelerometer (Actigraph − Model wGT3x + BT) on the non-dominant hand’s wrist using a non-removable strap. Participants were asked to wear the accelerometer until the follow-up visit (i.e., not taken off during sleeping hours). Raw accelerometer data were processed via an open-source R-package ‘GGIR’ (version 1.11-0) [33] to calculate physical activity and sleep variables on each day: total time (bouted and unbouted) spent engaging in moderate- to vigorous-intensity physical activity (MVPA), light intensity physical activity (LPA), sedentary behavior, and sleep hours. Euclidean Norm Minus One was used as the accelerometer metric to define LPA and MVPA, with the cut-offs being 25 and 100 milli-gravitational (mg) units, respectively [33]. Because we used wrist-worn accelerometers, it was impossible to determine posture and distinguish sedentary behavior from other types of inactivity. Hence, we used inactivity as a proxy for sedentary behavior. Only days with at least 16 h of wear time were included in the analysis.

### 2.4. Statistical Analyses

Baseline characteristics were summarized using mean (SD) for continuous variables and count (percentages) for categorical variables. For the derived daily glucose outcomes, nutrient intakes, physical activity, and sleep variables, only data from the 7-day study period (Day 4 to Day 10) were used. 

We used generalized estimating equations (GEE) models with one covariate at a time to assess associations of baseline characteristics, nutrient intakes, physical activity, sedentary behavior, and sleep duration with each of the glucose outcomes. The associations were expressed as the regression coefficients and the 95% confidence intervals (CI). The GEE models used daily glucose outcomes as dependent variables and participants as clusters. Because there was no clear evidence indicating specific day-to-day correlation patterns within each individual for the continuous glucose metrics, we chose an independent within-individual correction structure, to preserve mores statistical power. Sensitivity analysis was conducted using first-order autoregressive (AR1) correlation structure, but this did not substantially change the results. We also used GEE models adjusting for BMI and MVPA as these were the strongest lifestyle-related predictors of glucose metrics. Because results were essentially the same after additional adjustment for education level, we did not include this covariate in the multivariable models. A *p*-value < 0.05 was considered statistically significant. All the analyses were performed in R (version 3.6.1). The R package ‘geepack’ was used for GEE models. 

## 3. Results

### 3.1. Participants’ Characteristics

We recruited 33 individuals, but five were excluded from the analyses: one did not fulfill the inclusion criteria, three withdrew because of sensor discomfort, and one had surgery before study commencement. Data of the remaining 28 participants were included in the data analysis. Table 1 summarizes the baseline characteristics of study participants. Most participants were males (64%), and they were of Chinese (54%), Indian (25%), or Malay (21%) ethnicity. About 32% reported having a university degree. The mean age was 46.0 (SD 9.9) years, the mean BMI was 27.51 (SD 1.76) kg/m^2^, and the mean HbA1c was 5.47% (SD 0.40). On average, the participants provided 5.7 (SD 0.9) days of valid accelerometer data, 6.4 (SD 1.3) days of valid glucose monitoring data, and 6.1 (SD 1.3) days of valid dietary data per person during the 7-day study period for the analysis (Supplemental Material Table S1). The average measurement hours per day during the valid days were 23.8 (SD 0.7) hours for the accelerometer and 21.8 (SD 1.8) hours for the glucose monitoring. During the 7-day study period, there were 179 participant-days with valid CGM data, of which 145 participant-days had valid accelerometer data and 142 participant-days had valid dietary data. 118 participant-days had all three types of valid data available simultaneously.

### 3.2. Associations of Demographics and Metabolic Measures with Glucose Metrics

Table 2 shows the associations between baseline characteristics and glucose metrics throughout the seven days. Age, sex, and ethnicity were not significantly associated with any of the measures of glucose variation. Having a university degree was associated with a higher mean glucose level (β = 0.44, *p* = 0.04) and less time-in-range (β = −4.38, *p* = 0.02) as compared to those with a low education level. A higher BMI (β = 0.12 per kg/m^2^, *p* = 0.01) and body fat (β = 0.03 per kg, *p* = 0.01) were associated with a higher mean glucose level. In addition, a higher BMI was associated with less variation in glucose levels as measured by the CV% (β = −0.85 per kg/m^2^, *p* = 0.049) and more time-in-range (β = 1.66, *p* = 0.03). This result was due to an association between higher BMI and less time below range as BMI was not associated with time above range (Supplemental Material Table S2). 

Fasting glucose was inversely associated with CV (β = −5.03 per mmol/L, *p* = 0.04). We also observed significant associations between the baseline MMTT measurements and glucose levels throughout the following week. A higher glucose and insulin iAUC and a lower Matsuda insulin sensitivity index were associated with higher mean glucose levels, obtained from glucose monitoring. In addition, a higher 2-h glucose level and glucose, and insulin iAUC during the MMTT were associated with a higher percentage time above range (Supplemental Material Table S2). No significant associations were found between HOMA2-IR, the insulinogenic index, or the disposition index glucose metrics. 

### 3.3. Associations between Lifestyle Behaviors and Glucose Metrics 

Table 3 shows the associations between lifestyle behaviors and glucose monitoring measures. Neither movement behaviors nor dietary intakes were associated with mean glucose levels. However, MVPA was associated with lower glycemic variability as assessed by the CV%, and this association became stronger after adjustment for BMI (β = −3.03 per hr/d, *p* < 0.001). Sedentary behavior was associated with a higher CV, but this association was no longer significant after adjustment for MVPA and BMI. Sleep duration and light intensity physical activity were not significantly associated with variation in glucose levels. 

Of the dietary measures, PUFA intake was associated with a lower glucose CV% after adjustment for MVPA and BMI (β = −2.23 per 5 en%, *p* < 0.001). Furthermore, higher PUFA (multivariable β = 3.21 per 5 en%, *p* = 0.02) and protein intake (β = 0.90, *p* = 0.007) were associated with more time-in-range, whereas higher carbohydrate intake was associated with less time-in-range (β = −0.59, *p* = 0.04) (Table 3). 

## 4. Discussion

We continuously monitored dietary intake, movement behaviors, and glucose levels over seven days to examine determinants of glycemic variability in persons at high risk of diabetes. A higher BMI, greater body fatness, and higher glucose and insulin levels during the mixed meal tolerance test were associated with higher mean glucose levels during the week. Moderate- to vigorous-intensity physical activity and higher PUFA intake were associated with lower glycemic variability and more time in the desired glucose range. In addition, higher protein intake and lower carbohydrate intake were associated with more time-in-range. These findings suggest that diet composition and physical activity behaviors are important determinants of variation in glucose levels throughout the day in persons at risk of diabetes. 

We observed that more moderate- to vigorous-intensity physical activity was associated with lower glycemic variability. Experimental studies have shown that exercise or muscle contraction lead to increased concentrations of GLUT4 in the cell plasma membrane and increases insulin-dependent glucose uptake [34], which may reduce glycemic variability [10]. Although more light-intensity physical activity and less sedentary behavior also tended to be associated with less glycemic variation, these associations were not significant after multivariable adjustment. This suggests that physical activity of at least moderate intensity may be more effective for improving glucose control than activities of lighter intensity. However, these results require confirmation in studies with larger sample size. Although it is well-documented that physical activity can improve fasting blood glucose and HbA1c levels [11,35], studies of physical activity and glycemic variability have only focused on persons with diabetes [36,37,38,39]. In a meta-analysis of 11 studies, exercise significantly reduced average glucose levels and time spent in hyperglycemia in persons with T2DM, as measured by CGM [37]. In a recent study, higher total physical activity was associated with lower average postprandial glucose areas-under-the-curve in persons with type 1 diabetes [39]. Our study extends the evidence on the impact of physical activity on variation in glucose levels to persons without diabetes. 

We observed that higher PUFA intake was associated with lower glycemic variability. Several underlying biological mechanisms may explain this putative beneficial effect of PUFA on glucose metabolism. PUFAs may improve insulin sensitivity and improve insulin secretion by protecting pancreatic beta cells from damage induced by free radicals [40]. Vegetable oils high in PUFAs improved glycemic regulation in persons with T2DM in several trials, although this finding was not consistent [41]. In a meta-analysis of trials, PUFAs significantly improved insulin secretion capacity and lowered fasting glucose levels in older individuals and those with diabetes [42]. In addition, PUFA intake has been associated with a lower risk of T2DM in several prospective cohort studies [43]. Our findings suggest that higher PUFA intake may reduce glucose variability in persons without diabetes, but this requires confirmation in future studies. We also observed that higher PUFA and protein intake were associated with greater time-in-range, whereas higher carbohydrate intake was associated with lower time-in-range. Similarly, in a study in persons with type 1 diabetes, higher protein intake was associated with reduced glycemic variability, as measured by the mean amplitude of glycemic excursions (MAGE) and CV. Besides, lower carbohydrate intake was associated with lower nocturnal continuous overlapping net glycemic action (CONGA) values [44]. 

In our study, higher glucose and insulin levels during the mixed meal tolerance test were associated with higher mean glucose levels throughout the week and more time above the desired glucose range. In contrast, HbA1c levels were not significantly associated with mean glucose levels. HbA1c is an index of average blood glucose measurement over several months, whereas continuous glucose monitoring captures rapid changes and may facilitate understanding of dynamic glycemic patterns [3,45,46]. 

A higher BMI and more body fat were associated with higher mean glucose levels throughout the week. A higher BMI was also associated with lower glycemic variability and more time-in-range. However, this only reflected less time below the desired range which was probably due to the higher mean glucose levels in participants with a higher BMI. In a US study of persons without diabetes, morbid obesity (BMI ≥ 40 kg/m^2^) was associated with greater glycemic variability than having a normal weight [47]. Because our study did not include participants with morbid obesity, our findings cannot be directly compared with this study, and additional studies are needed on the association between body composition and glycemic variation in persons without diabetes. 

A strength of this study was the use of flash glucose monitoring for seven days and an accelerometer to measure movement objectively. We used a custom-built mobile dietary record app incorporating a local food database to collect information on dietary intakes to reduce the recall bias of food entry. However, several study limitations should be considered. First, our sample size was modest, possibly limiting our statistical power to detect subtle effects of some of the examined determinants on glucose metrics. Further research using larger sample sizes is needed. Second, as in any observational study, we cannot fully exclude the possibility of residual confounding. In particular, confounding by other correlated dietary factors is possible and requires further research. Third, wearing accelerometers and recording food consumption may have affected these lifestyle behaviors. However, the risk of exposure misclassification would be unlikely to be differential because participants were not aware of their variation in blood glucose levels. Fourth, the glucose monitor readings from flash glucose monitoring devices can be lower than venous blood or capillary blood glucose measurements [48,49]. However, these devices allow comparatively accurate assessment of trends in glucose values [28]. Fifth, although we conducted end-user evaluation, and subsequent refinements, we were unable to conduct a comprehensive validation of the digital food record component of the app. However, food records are established and well-accepted methods to assess dietary intake. Although digital food records represent a different medium than paper-based records, it is not a new method of dietary assessment and no difference in reported intakes between paper-based and digital records was observed in previous research [50]. Furthermore, average intakes of macronutrients in our study were similar to estimated intakes in the Singapore National Nutrition Survey [51].

## 5. Conclusions

We showed that moderate- to vigorous-intensity physical activity and higher PUFA intake were associated with lower variation in glucose levels in persons at a higher risk of diabetes. In addition, higher protein and polyunsaturated fat intake and lower carbohydrate intake were associated with more time-in-range. These findings suggest that physical activity and dietary composition can contribute to fewer fluctuations in glucose levels throughout the day, which may represent an additional pathway to improve cardio-metabolic health [5,6,7,8]. More research on the impact of lifestyle interventions on variation in glucose levels in persons at a higher risk of diabetes and its long-term health impact is warranted.

## Figures and Tables

**Table 1:** Characteristics of study participants (*n* = 28).

	*n* (%) or Mean (SD)
Baseline Characteristics	
Sex	
Female	10 (36%)
Male	18 (64%)
Ethnicity	
Chinese	15 (54%)
Indian	7 (25%)
Malay	6 (21%)
Age (years)	46.0 (9.9)
Education	
Below A’ level or the equivalent	10 (35.7%)
A’ level or the equivalent	9 (32.1%)
University	9 (32.1%)
Body mass index (kg/m^2^)	27.5 (1.8)
Body fat (kg)	25.8 (5.7)
Fat free mass (kg)	51.4 (9.4)
HbA1c (%)	5.5 (0.4)
Fasting glucose, (mmol/L)	4.8 (0.3)
HOMA2-IR	1.1 (0.5)
Accelerometer measures	
Moderate- to vigorous-intensity physical activity (hrs/d)	1.6 (0.6)
Light intensity physical activity (hrs/d)	4.9 (1.4)
Sedentary (hrs/d)	11.0 (1.7)
Sleep (hrs/d)	5.4 (1.1)
Diet intakes	
Protein (en%)	17.6 (4.4)
Saturated fat (en%)	13.0 (1.8)
Monounsaturated fat (en%)	11.5 (1.8)
Polyunsaturated fat (en%)	5.9 (1.2)
Carbohydrates (en%)	50.3 (6.9)
Fiber (g/1000 kcal)	9.7 (3.6)
Measures of glucose variation	
Mean glucose (mmol/L)	4.8 (0.5)
SD glucose (mmol/L)	0.9 (0.3)
%CV glucose	19.2 (4.9)
%Time-in-range (3.0–7.8 mmol/L)	95.4 (7.1)
%Time above range (>7.8 mmol/L)	2.3 (3.5)
%Time below range (<3.0 mmol/L)	2.4 (6.5)

SD, standard deviation; HbA1c, hemoglobin A1c; HOMA2-IR, homeostasis model assessment of insulin resistance; CV, coefficient of variation.

**Table 2:** Associations between baseline characteristics and glucose monitoring metrics over seven days.

	Mean Glucose		%CV Glucose		%Time-in-Range (3.0–7.8 mmol/L)	
	Estimate (CI)	*p*	Estimate (CI)	*p*	Estimate (CI)	*p*
Sex						
Female	Reference		Reference		Reference	
Male	0.14 (−0.27, 0.55)	0.51	−2.33 (−6.06, 1.40)	0.22	1.12 (−4.13, 6.38)	0.68
Ethnicity						
Chinese	Reference		Reference		Reference	
Indian	−0.14 (−0.68, 0.39)	0.60	0.70 (−3.74, 5.14)	0.76	−2.13 (−7.50, 3.24)	0.44
Malay	−0.27 (−0.85, 0.31)	0.36	1.50 (−3.18, 6.17)	0.53	−5.32 (−15.07, 4.43)	0.28
Age ^a^ (years)	−0.05 (−0.27, 0.17)	0.68	−0.06 (−1.67, 1.55)	0.94	−1.92 (−4.87, 1.04)	0.20
Education						
Below A’ level or the equivalent	Referent					
A’ level or the equivalent	0.20 (−0.31, 0.71)	0.44	−0.07 (−0.32, 0.18)	0.59	−2.26 (−6.70, 2.19)	0.32
University	0.44 (0.03, 0.86)	0.04	−0.13 (−0.32, 0.07)	0.20	−4.38 (−8.10, −0.66)	0.02
BMI (kg/m^2^)	0.12 (0.03, 0.22)	0.01	−0.85 (−1.69, −0.00)	0.049	1.66 (0.17, 3.14)	0.03
Body fat (kg)	0.03 (0.01, 0.05)	0.01	0.06 (−0.25, 0.37)	0.69	−0.12 (−0.45, 0.22)	0.50
Fat free mass (kg)	0.01 (−0.02, 0.03)	0.62	−0.11 (−0.26, 0.05)	0.17	0.15 (−0.03, 0.33)	0.10
HbA1c (%)	0.32 (−0.13, 0.78)	0.16	−1.21 (−6.88, 4.46)	0.68	−3.03 (−10.01, 3.96)	0.40
Fasting glucose, (mmol/L)	0.37 (−0.28, 1.02)	0.27	−5.03 (−9.78, −0.27)	0.04	−2.55 (−12.20, 7.10)	0.61
HOMA2−IR	0.35 (−0.03, 0.74)	0.072	−0.18 (−4.32, 3.96)	0.932	−0.29 (−4.71, 4.14)	0.898
2-h glucose, (mmol/L)	0.17 (−0.04, 0.39)	0.11	0.84 (−1.32, 3.00)	0.44	−2.47 (−5.32, 0.37)	0.09
Glucose iAUC (1000 units)	2.05 (0.44, 3.65)	0.01	11.56 (−5.52, 28.64)	0.19	−7.06 (−29.09, 14.97)	0.53
Matsuda Index	−0.05 (−0.08, −0.01)	0.02	−0.04 (−0.49, 0.42)	0.87	0.21 (−0.28, 0.71)	0.40
Insulin iAUC (1000 units)	0.14 (0.08, 0.20)	<0.001	0.45 (−0.55, 1.46)	0.38	−0.73 (−1.82, 0.36)	0.19
Insulinogenic index (1000 units)	8.13 (−7.12, 23.38)	0.30	−37.65 (−218.22, 142.92)	0.68	−90.05 (−332.14, 152.05)	0.47
Disposition index	−0.45 (−1.02, 0.12)	0.12	1.75 (−3.63, 7.13)	0.52	2.62 (−4.95, 10.19)	0.50

^a^ Regression coefficients are expressed per 10-year increment. CI, confidence interval; CV, coefficient of variation; BMI, body mass index; HbA1c, hemoglobin A1c; HOMA2, homeostasis model assessment of insulin resistance; iAUC, incremental area under curve.

**Table 3:** Associations between movement behaviors, dietary intake, and glucose monitoring metrics over seven days.

	Mean Glucose		%CV Glucose		%Time-in-Range (3.0–7.8 mmol/L)	
	Estimate (CI)	*p*	Estimate (CI)	*p*	Estimate (CI)	*p*
Movement behaviors						
Moderate-to-vigorous intensity physical activity (hrs/d)	−0.07 (−0.23, 0.09)	0.39	−1.77 (−3.09, −0.46)	0.008	0.94 (−0.93, 2.81)	0.32
Multivariable-adjusted	0.05 (−0.15, 0.25)	0.64	−3.03 (−4.67, −1.39)	<0.001	2.61 (0.38, 4.84)	0.02
Light intensity physical activity (hrs/d)	−0.05 (−0.15, 0.05)	0.36	−0.70 (−1.64, 0.24)	0.14	0.09 (−1.04, 1.22)	0.88
Multivariable-adjusted	−0.04 (−0.13, 0.05)	0.35	−0.42 (−1.32, 0.47)	0.36	−0.15 (−1.40, 1.09)	0.81
Sedentary (hrs/d)	0.00 (−0.06, 0.06)	0.96	0.56 (0.03, 1.08)	0.04	−0.35 (−1.01, 0.31)	0.31
Multivariable-adjusted	−0.02 (−0.08, 0.04)	0.54	0.42 (−0.17, 1.01)	0.16	−0.33 (−1.03, 0.37)	0.36
Sleep (hrs/d)	0.01 (−0.09, 0.12)	0.83	−0.03 (−1.06, 0.99)	0.95	0.38 (−1.01, 1.76)	0.60
Multivariable-adjusted	0.02 (−0.06, 0.11)	0.63	−0.27 (−1.06, 0.52)	0.51	0.62 (−0.38, 1.62)	0.23
Diet measures ^a^						
Protein (en%)	−0.00 (−0.09, 0.09)	0.98	−0.07 (−0.65, 0.50)	0.80	1.04 (0.22, 1.86)	0.01
Multivariable-adjusted	−0.03 (−0.09, 0.03)	0.32	−0.31 (−0.79, 0.18)	0.22	0.90 (0.25, 1.56)	0.007
Saturated fat (en%)	0.06 (−0.11, 0.22)	0.52	0.37 (−0.79, 1.53)	0.53	0.13 (−2.35, 2.60)	0.92
Multivariable-adjusted			0.52 (−0.64, 1.67)	0.38	0.19 (−2.34, 2.71)	0.89
Monounsaturated fat (en%)	0.01 (−0.17, 0.20)	0.90	−0.61 (−1.78, 0.55)	0.30	1.14 (−1.23, 3.51)	0.35
Multivariable-adjusted	−0.03 (−0.22, 0.16)	0.78	−0.44 (−1.89, 1.01)	0.55	0.90 (−1.56, 3.35)	0.47
Polyunsaturated fat (en%)	−0.01 (−0.19, 0.17)	0.91	−1.64 (−3.40, 0.11)	0.07	2.39 (−0.84, 5.62)	0.15
Multivariable-adjusted	−0.01 (−0.18, 0.17)	0.94	−2.23 (−3.51, −0.94)	<0.001	3.21 (0.47, 5.94)	0.02
Carbohydrates (en%)	−0.00 (−0.05, 0.05)	0.91	0.11 (−0.29, 0.50)	0.60	−0.58 (−1.26, 0.09)	0.09
Multivariable-adjusted	0.01 (−0.03, 0.05)	0.58	0.20 (−0.19, 0.60)	0.31	−0.59 (−1.17, −0.02)	0.04
Fiber (g/1000 kcal)	0.04 (−0.07, 0.15)	0.46	−0.44 (−1.90, 1.01)	0.55	0.39 (−0.78, 1.57)	0.52
Multivariable-adjusted	0.003 (−0.10, 0.11)	0.96	0.08 (−1.11, 1.27)	0.89	−0.33 (−1.47, 0.80)	0.57

^a^ Regression coefficients are expressed per 5 percent of energy intake for nutrients and per 5 g/1000 kcal for fiber. Multivariable-adjusted models were adjusted for BMI and moderate- to vigorous-intensity physical activity. CV, coefficient of variation; CI, confidence interval.

## Data Availability

Requests for data can be emailed to the corresponding author.

## Supplementary Materials

The following supporting information can be downloaded at: https://www.mdpi.com/article/10.3390/nu14020366/s1, Table S1: Data on completeness of accelerometer and glucose monitor wear time and recording of dietary intake on the mobile phone app, Table S2: Baseline characteristics, movement behaviors, and dietary intakes in relation to the percentage of time below and above the glucose range over seven days.
