# Supplementary material for: Diet and Physical Activity as Determinants of Continuously Measured Glucose Levels in Persons at High Risk of Type 2 Diabetes
Source: Nutrients. 2022 Jan 15;14(2):366. doi: 10.3390/nu14020366 (PMC8781180; doi:10.3390/nu14020366)
Supplement: Supplementary file 1 [file nutrients-14-00366-s001.zip › nutrients-1523948-supplementary.pdf]

Supplemental Table S1. Data on completeness of accelerometer and glucose monitor wear time and recording of dietary intake on the mobile phone app

	**7-day study period**	**7-day study period with valid CGM measures**
**Accelerometer**		
Number of days with data	5.9 (0.4)	5.4 (1.1)
Number of days with valid data	5.7 (0.9)	5.2 (1.3)
**CGM**		
Number of days with data	6.9 (0.4)	6.4 (1.3)
Number of days with valid data	6.4 (1.3)	6.4 (1.3)
Measurement hours per day during the valid days: mean (SD)	21.8 (1.8)	21.8 (1.8)
**EMA (food)**		
Number of days with data*	6.8 (0.5)	6.2 (1.4)
Number of days with valid data (energy intakes lower than 500 kcal or higher than 5000 kcal)**	6.1 (1.3)	5.9 (1.5)

Data are mean number of days per person (SD)
* among the 26 participants who provided dietary data.
** among the 25 participants who provided dietary data.

Supplemental Table S2. Baseline characteristics, movement behaviors, and dietary intakes in relation to the percentage of time below and above the glucose range over seven days

	%Time below range (< 3.0 mmol/L)		%Time above range (> 7.8 mmol/L)	
	Estimate (CI)	*P*	Estimate (CI)	*P*
**Baseline characteristics**				
Sex				
Female	Reference		Reference	
Male	-0.20 (-4.93, 4.52)	0.93	-0.92 (-3.93, 2.09)	0.55
Ethnicity				
Chinese	Reference		Reference	
Indian	1.12 (-2.85, 5.10)	0.58	1.01 (-3.38, 5.40)	0.65
Malay	4.67 (-5.45, 14.80)	0.37	0.65 (-2.18, 3.48)	0.65
Age (years)[a]	2.23 (-0.75, 5.22)	0.14	-0.31 (-1.51, 0.88)	0.61
Education				
Below A level or the equivalent	Reference		Reference	
A level or the equivalent	1.46 (-5.94, 8.86)	0.70	0.54 (-2.99, 4.06)	0.77
University	-2.85 (-6.04, 0.34)	0.08	-0.45 (-2.75, 1.85)	0.70
BMI (kg/m^2)	**-1.66 (-3.20, -0.12)**	**0.04**	0.00 (-0.37, 0.38)	0.98
Body fat (kg)	-0.04 (-0.14, 0.06)	0.44	0.16 (-0.15, 0.46)	0.31
Fat free mass (kg)	-0.12 (-0.29, 0.04)	0.14	-0.03 (-0.15, 0.10)	0.68
HbA1c (%)	0.38 (-4.79, 5.56)	0.88	2.64 (-2.00, 7.29)	0.27
Fasting glucose (mmol/L)	3.84 (-5.40, 13.08)	0.42	-1.29 (-5.51, 2.93)	0.55
HOMA2-IR	-1.89 (-4.06, 0.28)	0.09	2.18 (-1.41, 5.78)	0.23
2-hr glucose (mmol/L)	1.22 (-1.65, 4.09)	0.41	**1.26 (0.12, 2.40)**	**0.03**
Glucose iAUC (1000 units)	-10.50 (-29.45, 8.46)	0.28	**17.56 (6.21, 28.91)**	**0.002**
Matsuda Index	0.05 (-0.34, 0.44)	0.81	-0.26 (-0.58, 0.05)	0.10
Insulin iAUC (1000 units)	-0.43 (-0.98, 0.13)	0.13	**1.16 (0.33, 1.98)**	**0.01**
Insulinogenic index (1000 units)	40.09 (-146.03, 226.20)	0.67	49.96 (-116.37, 216.29)	0.56
Disposition index	-1.63 (-8.35, 5.10)	0.64	-0.99 (-5.34, 3.35)	0.65
**Movement behaviors[b]**				
Moderate-to-vigorous intensity physical activity (hrs/day)	**-1.90 (-3.54, -0.26)**	**0.02**	-0.71 (-2.18, 0.76)	0.35
Light intensity physical activity (hrs/day)	0.30 (-0.74, 1.34)	0.57	-0.15 (-0.63, 0.33)	0.54
Sedentary (hrs/day)	0.41 (-0.34, 1.16)	0.28	-0.09 (-0.44, 0.27)	0.63
Sleep (hrs/day)	-0.71 (-1.83, 0.42)	0.22	0.09 (-0.33, 0.50)	0.68
**Diet measures[a,b]**				
Protein (en%)	-0.43 (-0.86, -0.00)	0.05	-0.47 (-1.04, 0.10)	0.10
Saturated fat (en%)	-1.26 (-3.43, 0.90)	0.25	1.08 (-0.82, 2.97)	0.27
Monounsaturated fat (en%)	-1.31 (-2.91, 0.30)	0.11	0.41 (-1.80, 2.62)	0.72
Polyunsaturated fat (en%)	-2.41 (-5.14, 0.31)	0.08	-0.79 (-1.79, 0.20)	0.12
Carbohydrates (en%)	0.51 (-0.00, 1.03)	0.05	0.08 (-0.28, 0.43)	0.67

Fiber (g/1000 kcal)	0.41 (-0.26, 1.09)	0.23	-0.08 (-1.00, 0.83)	0.86

[a] Regression coefficients are expressed per 10 years for age, per 5 percent of energy for nutrients, and per 5 g/1000 kcal for fiber

[b]Estimates for movement and dietary behaviors were adjusted for moderate-to-vigorous intensity physical activity and BMI.

CI, confidence interval; BMI, body mass index; HbA1c, hemoglobin A1c; HOMA-IR, homeostasis model assessment of insulin resistance; iAUC, incremental area under curve
